# Supplementary material for: Diabetes self-care intervention strategies and their effectiveness in Sub-Saharan Africa: A systematic review
Source: PLoS One. 2024 Oct 15;19(10):e0305860. doi: 10.1371/journal.pone.0305860 (PMC11478876; doi:10.1371/journal.pone.0305860)
Supplement: S1 File — (DOCX) [file pone.0305860.s002.docx]

**Supplementary 1**

**:** JBI checklist of randomized controlled trials (RCT) critical appraise of methodological quality assessment tool result

| No | Items | (Ng'ang'a, 2022) | (Gathu, 2018) | (F. B. Hailu, Hjortdahl, P., & Moen, A, 2021) | (Webb, 2017) | (Tsobgny-Tsague, 2018) | (F. B. Hailu, Moen, A., & Hjortdahl, P., 2019) | (Leon, 2021) | (Farmer, 2021) | (E. O. Owolabi, Goon, D. T., & Ajayi, A. I. , 2019) | (Lamptey, 2023) | (Muchiri, 2016) | (Diriba, 2023) |
| --- | --- | --- | --- | --- | --- | --- | --- | --- | --- | --- | --- | --- | --- |
| 1 | Was true randomization used for assignment of participants to treatment groups? | Yes-1 | Yes-1 | Yes-1 | Yes-1 | Yes-1 | Yes-1 | No-0 | Yes-1 | Yes -1 | Yes-1 | Yes-1 | Yes-1 |
| 2 | Was allocation to treatment groups concealed? | No-0 | Yes-1 | Yes-1 | Yes-1 | Yes-1 | Yes -1 | Yes-1 | Yes-1 | No-0 | Yes-1 | Yes-1 | Yes-1 |
| 3 | Were treatment groups similar at the baseline? | Yes-1 | Yes-1 | Yes-1 | Yes-1 | Yes-1 | Yes1- | Yes-1 | Yes -1 | Yes-1 | Yes -1 | Yes -1 | Yes -1 |
| 4 | Were participants blind to treatment assignment? | No-0 | No-0 | No-0 | No-0 | Yes-1 | No-0 | No-0 | No-0 | Yes-1 | No-0 | No-0 | Yes-1 |
| 5 | Were those delivering treatment blind to treatment assignment? | Yes-1 | No-0 | No-0 | No-0 | No-0 | No-0 | No-0 | Yes-1 | No-0 | No-0 | No-0 | No-0 |
| 6 | Were outcomes assessors blind to treatment assignment? | Yes-1 | No-0 | No-0 | No-0 | Yes -1 | No-0 | No-0 | Yes-1 | No-0 | No-0 | Yes-1 | Yes-1 |
| 7 | Were treatment groups treated identically other than the intervention of interest? | Yes-1 | Yes-1 | No-0 | Yes-1 | Yes-1 | Yes-1 | Yes-1 | Yes-1 | Yes-1 | Yes -1 | No-0 | No-0 |
| 8 | Was follow up complete and if not, were differences between groups in terms of their follow up adequately described and analyzed? | Yes-1 | Yes-1 | Yes-1 | Yes-1 | Yes-1 | Yes-1 | Yes-1 | Yes-1 | Yes-1 | Yes-1 | Yes-1 | Yes-1 |
| 9 | Were participants analyzed in the groups to which they were randomized? | Yes-1 | Yes-1 | Yes-1 | Yes-1 | Yes-1 | Yes-1 | Yes-1 | Yes-1 | Yes-1 | Yes-1 | Yes-1 | Yes-1 |
| 10 | Were outcomes measured in the same way for treatment groups? | Yes-1 | Yes-1 | Yes -1 | No-0 | Yes-1 | Yes -1 | Yes -1 | Yes-1 | Yes-1 | Yes -1 | Yes-1 | Yes-1 |
| 11 | Were outcomes measured in a reliable way? | Yes-1 | Yes -1 | Yes-1 | No-0 | Yes-1 | Yes-1 | Yes-1 | Yes-1 | Yes-1 | Yes -1 | Yes-1 | Yes-1 |
| 12 | Was appropriate statistical analysis used? | Yes-1 | Yes-1 | Yes-1 | Yes-1 | Yes-1 | Yes-1 | Yes-1 | Yes-1 | Yes-1 | Yes -1 | Yes-1 | Yes-1 |
| 13 | Were the trial design appropriate and any deviations from the standard RCT design accounted for in the conduct and analysis of the trial? | Yes-1 | Yes-1 | Yes-1 | Yes-1 | Yes-1 | Yes-1 | Yes-1 | Yes-1 | Yes-1 | Yes -1 | Yes-1 | Yes-1 |
|  | 100% | 84 | 80 | 62 | 62 | 92 | 77 | 70 | 92 | 77 | 77 | 77 | 84 |
|  | Overall decision | Include | Include | Include | Include | Include | Include | Include | Include | Include | Include | Include | Include |

Key note, maximum 92%, minimum 62% and average assessment result 78%.

Supplementary 1 JBI checklist of quasi-experimental design (QE) critical appraise of methodological quality assessment tool result.

| No | Items | (Tamiru, 2023) |
| --- | --- | --- |
| 1 | Is it clear in the study what is the ‘cause’ and what is effect? | Yes-1 |
| 2 | Were the participants included in any comparison similar? | Yes-1 |
| 3 | Were participants included in any comparison receiving similar treatment/care, other than the exposure or intervention interest? | Yes -1 |
| 4 | Was there a control group? | Yes -1 |
| 5 | Were there multiple measurements of outcome both pre and post intervention? | Yes -1 |
| 6 | Was the follow up completed, if not were differences between groups in terms of their follow up adequately described? | Yes -1 |
| 7 | Were the outcomes of participants included in any comparison measured in the same way? | Yes -1 |
| 8 | Were outcomes measured in a reliable way | Yes -1 |
| 9 | Was appropriate statistical analysis used? | Yes -1 |
|  | Overall decision | Included=100 |
