# Supplementary material for: Diabetes self-care intervention strategies and their effectiveness in Sub-Saharan Africa: A systematic review
Source: PLoS One. 2024 Oct 15;19(10):e0305860. doi: 10.1371/journal.pone.0305860 (PMC11478876; doi:10.1371/journal.pone.0305860)
Supplement: Attachment — Submitted filename: Response Letter.docx [file pone.0305860.s004.docx]

Dear Editorial Team and Reviewers,

Thank you very much for comments and suggestions to make our manuscript scientifically rigorous. Below, we provided our responses to each reviewer’s points. If there are any unaddressed questions or suggestions, we are ready to further correction.

Thank you.

| No | Reviewer question | Response |
| --- | --- | --- |
|  | Reviewer 1 |  |
| 1 | How the SSA was included". In the search term the SSA does not exist. | Under method there is search strategy page 5 there is Sub-Saharan Africa (SSA) |
| 2 | Did the authors search for a particular author or not? Please provide some information about the level of literacy of the population. Does this affect the results. | We didn’t search author, our search strategy was guided by the search strategy outlined on page 5, and applying eligibility criteria to refine our selection, rather than focusing on authorship.  -Our study aimed not to assess the correlation between participant literacy levels and self-care strategy but to evaluate diabetes self-care intervention being utilized in SSA and their effectiveness. |
| 3 | Did the authors revise only abstract or full manuscripts. | If the question is about screening of article? Staring from title, abstract and the full text assessed to extract out important information |
|  | Reviewer #2: |  |
| 1 | The introduction section was good and from my side there is nothing to add. | Thank you very much for your encouragement. |
| 2 | • It seems that you are here assuming from the beginning that there will be a reduction in complication: "Outcomes included glycaemic control, knowledge and behavioural change, adherence, and reduced complications." |  |
| 3 | • It is not clear if you have chosen these inclusion criteria while you were selecting the papers, or you are just mentioning here what you have after you finished the screening. If you have selected these criteria from the beginning, then please justify using these outcomes. "Outcomes included glycaemic control, knowledge and behavioural change, adherence, and reduced complications." | Before we were screening the paper, we set criteria and based on criteria we selected papers. |
| 4 | •Why was the time frame set to be from 2013 and not before? | This is to get the most updated information on self-care intervention. |
| 5 | It is not clear how adherence could be among the outcomes and intervention: The chosen interventions were specifically cantered around diabetes self-care, with particular emphasis on aspects such as diet, glucose monitoring, medication adherence, physical activities, and psychosocial support. "Outcomes included glycemic control, knowledge and behavioral change, adherence, and reduced complications | Diabetes self-care interventions are pivotal in improving the outcomes such as glycaemic control, behavioural change, increases the adherences to self-care activities. Which intervention is effective in resource limited setting like SSA.  If effective interventions are added the outcome of the mentioned activities increase and which reduce the complication related to diabetes. |
|  | • In Data analysis, the parameters used to describe the data were not reported |  |
|  | • Using the year of publication as a factor to categorize the data seems irrelevant and adds no important information to the paper. | We removed from Table 1 |
|  | • Reporting the origin of the studies in the first section of the results is not needed. You can mention the two countries with the highest number of studies. Avoid repetition of data from the table. | We removed repeated data found in Table 1 |
|  | • The age of the participants (mean or range) is very important and relevant to the paper. | We update table 1 including mean age of participants |
|  | The interventions were defined differently in the inclusion criteria and in the results. In the inclusion criteria: "The chosen interventions were specifically cantered around diabetes self-care, with particular emphasis on aspects such as diet, glucose monitoring, medication adherence, physical activities, and psychosocial support." In the results: "In our systematic review, we identified three groups of interventions in Sub-Saharan African countries. These include diabetes self-management education provided by nurses and other healthcare providers, phone-based short messaging service (SMS) text messaging, and other specific interventions." | We corrected idea on page 6 and 9. |
|  | • There are no specific numbers used to describe the interventions' effectiveness. The comparison with the control group is unclear, and the significance of the difference was not reported. Using general terms to describe the effectiveness is not sufficient. Providing numbers for the control and intervention groups with relevant significance is essential to convey your point. | 38.5% were effective in either of the primary or secondary outcomes measured (page 10). |
|  | • Table 3 should contain the number of participants in the control and intervention groups for each study, the effectiveness for each primary and secondary outcome, and the significance of results. That would make the findings clearer instead of using general terms. | We corrected table 3. |
|  | • There is repetition in the method section: "Thirteen eligible studies published between 2013 and 2023 were reviewed. The review revealed diverse interventions, implementation strategies, durations, and their effectiveness in improving diabetes management within the region." The first section in the discussion should only summarize the results. | We corrected and removed repetition of idea. |
|  | • Repetition of citations is unnecessary in the discussion (Second discussion part). Instead, report the strategies found in your research without re-mentioning the citations. | We removed repeated citation and corrected according to the reviewer suggestions in page 9 and 10. |
|  | Avoid using general terms in the discussion. Instead, provide specific numbers to reflect the significance of the intervention's efficacy. For example, instead of saying, "a significant effect on reducing HbA1 levels," specify how much HbA1c was reduced and whether it was clinically relevant. | According to reviewers comment we removed general terms and replaced by specific and relevance words. |
|  | • The discussion contains many general terms that should be specified using numbers to make the argument specific and scientifically precise, such as:- "This finding is consistent with a similar study conducted in another region of Africa." - "However, our findings contrast with a study conducted in Egypt, where a 3-month SMS text messaging intervention showed a significant effect on reducing HbA1 levels and fostering self-management behaviour."  - "Family involvement in healthcare, especially diabetes, can significantly influence an individual's ability to adhere to treatment plans and make necessary lifestyle changes." | We corrected and highlighted.  Corrected as ‘’ However, our review findings on SMS text messaging contrast with a study conducted in Egypt, where SMS text messaging intervention showed a significant effect on reducing HbA1 levels by 1% and fostering self-management behavior in Egypt’’.  We corrected as ‘’In the present review, family-supported, culturally tailored interventions, especially when combined with education, demonstrated effective outcomes in reduction HbA1c by 1.1% among intervention group |
|  | • Delete repetitions in the paper to make the discussion robust and of high quality, such as: - "In our review, the provision of a glucose machine, along with training, logbooks for registration, and waste management materials, significantly enhanced the adherence to self-monitoring of blood glucose after a 6-month follow-up."  "The present systematic review demonstrated a notably high adherence to glucose self-monitoring due to the provision of glucose testing machines and the comprehensive training and support provided to the participants. This underscores the significance of supporting patients with diabetes, as it significantly improves adherence and consequently reduces complications related to diabetes." | We deleted repeated idea and corrected as  ‘’In this review, we observed increased in the adherence to self-monitoring of blood glucose up to 64.3% and reduction of mean HbA1c 7.96 to 7.07% among patients following the provision of glucose meters, training sessions, logbooks for record-keeping, and proper disposal mechanisms.’’ |
|  | • The conclusion is too long and should be summarized | According to reviewer suggestions, we summarized conclusion from 176 to 123 words count and highlighted. |
